# Stabilising Cobalt Sulphide Nanocapsules with Nitrogen-Doped Carbon for High-Performance Sodium-Ion Storage

**DOI:** 10.1007/s40820-020-0391-9

**Published:** 2020-02-12

**Authors:** Yilan Wu, Rohit R. Gaddam, Chao Zhang, Hao Lu, Chao Wang, Dmitri Golberg, Xiu Song Zhao

**Affiliations:** 1 School of Chemical Engineering, The University of Queensland, St Lucia, Brisbane, QLD 4072 Australia; 2 School of Chemistry, Physics and Mechanical Engineering, Science and Engineering Faculty, Queensland University of Technology, Brisbane, QLD 4001 Australia; 3 Institute of Materials for Energy and Environment, College of Materials Science and Engineering, Qingdao University, 308 Ningxia Road, Qingdao, 266071 People’s Republic of China

**Keywords:** Cobalt sulphide, Nitrogen-doped carbon, Core–shell structure, Sodium-ion capacitors

## Abstract

- Cobalt sulphide nanoparticles are encapsulated in nitrogen-rich carbon cages via a simple and scalable method.
- Insight into sodium storage mechanism is systematically studied via in situ TEM and XRD techniques.
- The sodium-ion capacitor device achieved high energy densities of 101.4 and 45.8 Wh kg^−1^ at power densities of 200 and 10,000 W kg^−1^, respectively, holding promise for practical applications.

**Electronic supplementary material:**

The online version of this article (10.1007/s40820-020-0391-9) contains supplementary material, which is available to authorized users.

## Abstract (article highlights)

Conversion-type anode materials with a high charge storage capability generally suffer from large volume expansion, poor electron conductivity, and sluggish metal ion transport kinetics. The electrode material described in this paper, namely cobalt sulphide nanoparticles encapsulated in carbon cages (Co_9_S_8_@NC), can circumvent these problems. This electrode material exhibited a reversible sodium-ion storage capacity of 705 mAh g^−1^ at 100 mA g^−1^ with an extraordinary rate capability and good cycling stability. Mechanistic study using the in situ transmission electron microscope technique revealed that the volumetric expansion of the Co_9_S_8_ nanoparticles is buffered by the carbon cages, enabling a stable electrode–electrolyte interface. In addition, the carbon shell with high-content doped nitrogen significantly enhances the electron conductivity of the Co_9_S_8_@NC electrode material and provides doping-induced active sites to accommodate sodium ions. By integrating the Co_9_S_8_@NC as negative electrode with a cellulose-derived porous hard carbon/graphene oxide composite as positive electrode and 1 M NaPF_6_ in diglyme as the electrolyte, the sodium-ion capacitor full cell can achieve energy densities of 101.4 and 45.8 Wh kg^−1^ at power densities of 200 and 10,000 W kg^−1^, respectively.

**Electronic supplementary material:**

The online version of this article (10.1007/s40820-020-0391-9) contains supplementary material, which is available to authorized users.

## Graphical abstract

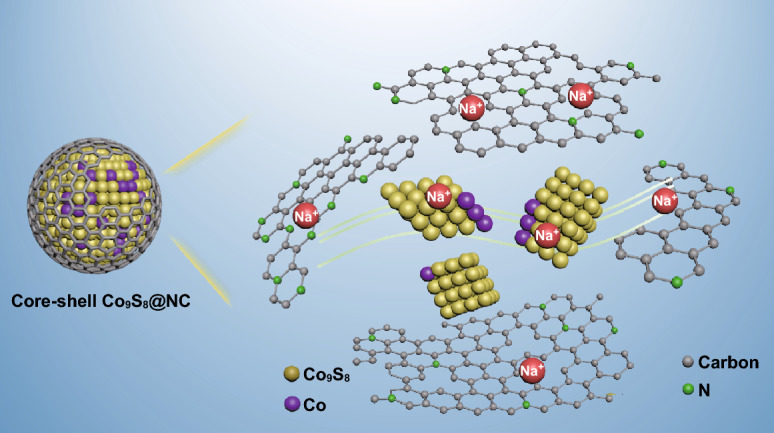

## Introduction

Sodium-ion batteries (NIBs) have resurfaced as the most promising energy storage technology for large-scale energy storage applications. With a wide spectrum of cathode materials suitable for NIBs, it has been of a great challenge for anode materials to compete with the current lithium-ion battery technology. Transition metal chalcogenides (TMCs) with high charge storage capacity, suitable redox voltage, and good electron conductivity have advantages compared to their oxide counterparts [1–3]. Among various TMCs, cobalt sulphides hold a great potential as anode materials for high-performance NIBs due to their high theoretical capacities, relatively low voltage plateau, and low cost [4–6]. Unfortunately, this family of materials suffers from sluggish kinetics of sodium-ion transport and large volume changes during charge/discharge, causing problems such as severe pulverisation and unstable solid electrolyte interphase (SEI) films [7].

Strategies have been proposed to address the above issues, including optimisation of electrode materials [8]. Nanoparticles (NPs) have been shown to not only improve the reaction kinetics due to shortened charge transport pathway but also effectively relieve mechanical strain induced by volume expansion [9]. However, NPs tend to aggregate during charge/discharge, leading to rapid loss in electroactivity. Dispersing NPs on carbon substrates has been shown to be a good solution to solving the particle aggregation problem [10]. Here we demonstrate a strategy for stabilising NPs by encapsulating them in carbon cages. Effective confinement from carbon shells not only eases the self-aggregation and pulverisation but also buffers the volumetric expansion and ensures a stable SEI film during sodiation/desodiation [11, 12]. Moreover, the enhanced contact between carbon layer and active components in the core–shell structure provides sufficient channels for fast electron/ion transport, thereby increasing the electronic conductivity and charge storage kinetics of the composite [13]. On the other hand, doping of carbon materials with heteroatoms such as nitrogen, sulphur, phosphorous, or boron can improve ionic and electronic conductivity [14–16]. Also, doping-induced defects on carbon could create localised active sites to accommodate sodium ions and favour ion transfer, giving rise to higher sodium storage performance [17, 18].

In this work, nitrogen-doped carbon frame was used to stabilise Co_9_S_8_ nanocapsules. The obtained electrode material (hereafter designated as Co_9_S_8_@NC) was used as anode for sodium-ion storage. It delivered a specific capacity as high as 705 mAh g^−1^ at 100 mA g^−1^ and exhibited an excellent rate performance (613 mAh g^−1^ at 4000 mA g^−1^), which is among the highest in all reported Co_9_S_8_ electrodes for NIBs [4, 5, 19–22]. Insight into sodium storage mechanism in Co_9_S_8_@NC is systematically studied and discussed via multiple analytical methods. The synthetic method is very versatile and can be easily extended to fabricate other TMC-based composites for energy storage.

## Experimental Section

### Synthesis of Co_9_S_8_@NC Composites

The Co_9_S_8_@NC nanocapsules were synthesised via a facile and scalable one-pot route. Typically, a certain amount of hydrate cobalt sulphate (CoSO_4_·7H_2_O) and 20 g melamine were dissolved in 20 mL deionised water with ultrasonication and stirring to obtain a pink suspension at room temperature. The suspension was centrifuged and collected, followed by being freeze-dried for 48 h. The resulting violet powder was annealed in a tube furnace at a rate of 5 °C min^−1^ to 750 °C and kept for 2 h under the nitrogen atmosphere to obtain the Co_9_S_8_@NC samples. Melamine was carbonised to form a carbon shell wrapping the Co_9_S_8_ nanocapsules. By varying the mass ratio of melamine/cobalt sulphate to be 40:6, 40:9, and 40:12, three samples denoted as Co_9_S_8_@NC-6, Co_9_S_8_@NC-9, and Co_9_S_8_@NC-12 were obtained. Because melamine contains rich nitrogen, high-content doping of nitrogen in the carbon shell occurred. For comparison, Co_9_S_8_ nanoparticles without carbon shell (Co_9_S_8_-NPs) were also prepared.

### Synthesis of Co_9_S_8_ Nanoparticles

As a reference, Co_9_S_8_ nanoparticles were also prepared by a modified method as previously reported [23]. Typically, 5 mmol CoSO_4_·7H_2_O and 5 mmol thiourea were dissolved in ethylene glycol (30 mL). The obtained solution was placed in a Teflon-lined stainless-steel autoclave and maintained at 160 °C for 12 h. After cooling down to the room temperature, the precipitates were collected and freeze-dried for 48 h, then followed by the same annealing treatment as the Co_9_S_8_@NC composites.

### Preparation of Cellulose-Derived Porous Carbon/Graphene Oxide Composite

Graphite oxide (GO) was prepared using the modified Hummers’ method [24, 25]. The resulting GO was dissolved in deionised (DI) water to form GO suspension (2 mg mL^−1^) by ultrasonication in ice bath for 1 h. Then 0.5 g MC powder and 1.5 g zinc chloride (ZnCl_2_) were added into 25 mL GO suspension with stirring for 1 h. Afterwards, the mixture was freeze-dried for 24 h, followed by annealing treatment at 550 °C for 2 h under nitrogen flow at a heating rate of 5 °C min^−1^. The obtained sample was washed with hydrogen chloride solution and DI water to remove residuals. After drying at 60 °C for 48 h, the cellulose-derived porous carbon/graphene oxide composite was obtained and denoted as CG.

## Results and Discussion

### Structural and Morphological Characterisation

**Fig. 1:**
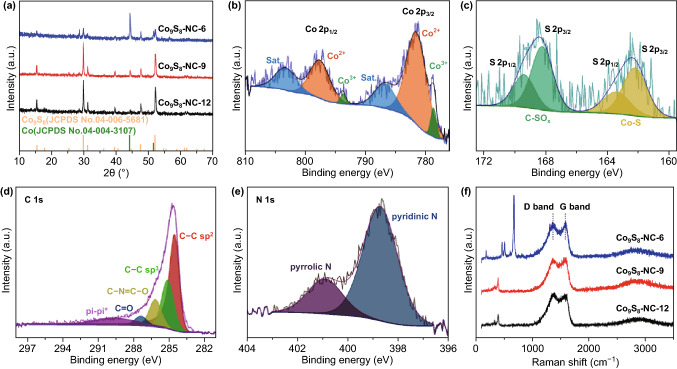
**a** XRD pattern of the Co_9_S_8_@NC composites and the standard XRD patterns of Co (JCPDS No. 04-004-3107) and Co_9_S_8_ (JCPDS No. 04-006-5681). **b-e** High-resolution XPS spectra of Co_9_S_8_@NC-9. **f** Raman spectra of the Co_9_S_8_@NC

The powder diffraction (XRD) patterns of the samples are shown in Fig. 1a. All major diffraction peaks of samples Co_9_S_8_@NC-9 and Co_9_S_8_@NC-12 can be indexed to cubic Co_9_S_8_ phase (JCPDS No. 04-006-5681) [26], indicating a complete conversion of CoSO_4_ to Co_9_S_8_. Also, the Co_9_S_8_@NC-6 and Co_9_S_8_@NC-9 sample show peaks at 44.2° and 51.6° corresponding to cubic cobalt (JCPDS No. 04-004-3107). Previous study has demonstrated that Co_9_S_8_ could be partially reduced to metallic cobalt through carbothermal reduction during annealing [27]. Therefore, the increase in melamine content in the precursor leads to the formation of higher amount of cobalt in the product.

The survey X-ray photoelectron spectroscopy (XPS) spectrum (Fig. S2) demonstrates the coexistence of Co, O, C, N, and S elements in the Co_9_S_8_@NC samples. The atomic ratio of each element is listed in Table S1. The XPS spectra of Co_9_S_8_@NC-9 were further analysed. In the Co 2*p*_3/2_ region, the peak located at 780.8 and 778.4 eV can be assigned to Co^2+^ and Co^3+^ (Fig. 1b) [28–30]. As shown in Fig. 1c, the characteristic peaks of Co-S located at 162.2 (S 2*p*_3/2_) and 163.4 eV (S 2*p*_1/2_), further confirming the presence of Co_9_S_8_. Moreover, binding energies at 168.2 (S 2*p*_3/2_) and 169.4 eV (S 2*p*_1/2_) correspond to C-SO_x_ groups, which may due to some SO_4_^2−^ residue on the sample. The typical high-resolution spectrum of C 1 s in Co_9_S_8_@NC is presented in Fig. 1d, which reveals the presence of C–C (*sp*^2^), C–C (*sp*^3^), C–N=C–O, C=O, and pi–pi* at binding energies of 284.6, 285.2, 286.2, 287.5, and 289.5 eV, respectively [18]. Also, the pi–pi* bond illustrates the existence of graphitic carbon in the sample. The nitrogen doping into the carbon shell can be verified by the high-resolution N 1 s spectrum shown in Fig. 1e, in which peaks of pyridinic N (398.7 eV) and pyrrolic N (400.9 eV) can be observed [31, 32]. The elemental content of nitrogen species on carbon is as high as 13.3% in the Co_9_S_8_@NC-9 sample. Large quantities of extrinsic defects can be introduced into carbon framework by pyridinic/pyrrolic nitrogen doping, hence favours ion transfer, and enhances the interaction property with sodium ions [17, 33, 34].

Raman spectra of Co_9_S_8_@NC samples are presented in Fig. 1f. The D peak (~ 1360 cm^−1^) arises from defect-activated in-plane breathing modes, corresponding to *sp*^3^ carbon bonding. The G peak (~ 1580 cm^−1^) is related to in-plane optical phonon modes and corresponds to *sp*^2^ carbon bonding. The 2D peak (~ 2700 cm^−1^) arises from a two-phonon process that is sensitive to the electronic structure. Raman spectra of the Co_9_S_8_@NC samples show *I*_D_/*I*_G_ values of ~ 1.0, indicating a high degree of defects due to nitrogen doping [16, 35]. In addition, for the Co_9_S_8_@NC-9 and Co_9_S_8_@NC-12 samples, the Raman bands below 750 cm^−1^ are well index to Co_9_S_8_ [23]. While in the Co_9_S_8_@NC-6 sample, the sharp and strong Raman shifts below 750 cm^−1^ are attributed to Co–Co stretching mode, indicating the presence of a large amount of metallic cobalt [36]. The porous structure feature of the Co_9_S_8_@NC composites was verified by the nitrogen adsorption–desorption measurement, as shown in Fig. S3a. The isotherms exhibit typical type-IV characteristics, implying a rich existence of mesopores in the Co_9_S_8_@NC samples. Correspondingly, the Brunauer–Emmett–Teller surface areas for Co_9_S_8_@NC-6, Co_9_S_8_@NC-9, and Co_9_S_8_@NC-12 were calculated to be 62.3, 42.1, and 19.6 m^2^ g^−1^, respectively, which are higher than that of Co_9_S_8_-NPs (15.1 m^2^ g^−1^). The average pore size of the Co_9_S_8_@NC-9 sample (Fig. S3b) shows the pore size is primarily distributed in the range of 2–4 nm. The presence of both micropores and mesopores can form a multichannel structure that facilitates the electrolyte penetration and electron/ion diffusion [37].

**Fig. 2:**
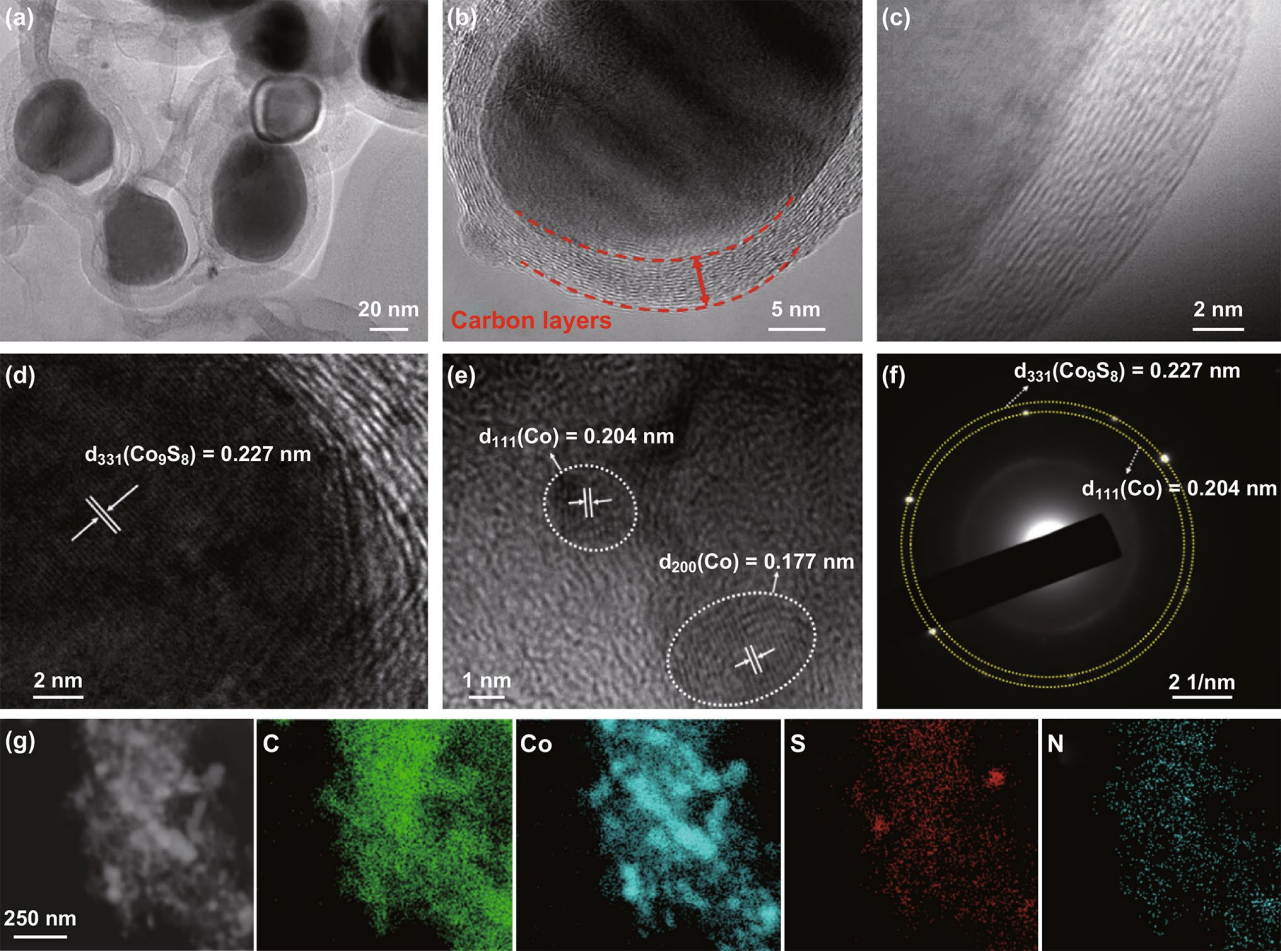
Morphology and structure of the as-prepared Co_9_S_8_@NC-9. **a**–**c** TEM images with different magnifications. **d**, **e** HRTEM images presenting the lattice fringes. **f** SAED pattern. **g** Elemental mapping images showing the distribution of C, Co, S, and N elements

Field emission scanning electron microscopy (FESEM) images of the Co_9_S_8_@NC composites are shown in Fig. S4. A panoramic view of the Co_9_S_8_@NC-6 and Co_9_S_8_@NC-9 shows the high yield of quasi-spherical NPs with a uniform size of about 100 nm, whereas the Co_9_S_8_@NC-12 sample exhibits irregular shapes. The transmission electron microscopy (TEM) images (Figs. 2a, b and S5) further demonstrate that the Co_9_S_8_@NC-9 exhibits the most intact core–shell structure with Co_9_S_8_ core NPs encapsulated and linked together by carbon sheets. A close observation (Fig. 2c) indicates the carbon shell consists of ~ 10–20 carbon layers that have a well-defined graphite crystalline structure. High-resolution TEM (HRTEM) image (Fig. 2d) evidences the good crystallisation of Co_9_S_8_ core. The continuous lattice fringe of ~ 0.227 nm corresponds to the (331) facet of cubic Co_9_S_8_, which is in line with the selected area electron diffraction (SAED) pattern results. Moreover, a small amount of ultrafine Co NPs (marked in circles) with ~ 2–5 nm in size can be observed, as shown in Fig. 2e, the presence of which has been demonstrated to increase the electronic conductivity of the composite by creating heterointerfaces and promote the formation of robust graphitic carbon [4, 38]. In addition, TEM elemental mapping analysis confirms the even distribution of Co, C, S, and N as the principal elemental components throughout the Co_9_S_8_@NC-9 sample, consistent with the full survey XPS spectrum result above. Such structure design offers multiple merits for achieving excellent sodium-ion storage. The direct, intimate contact between the core Co_9_S_8_ NPs and thin carbon shells provides efficient electron/ion transport media, which contribute to the excellent rate capability. The space-confined effect arising from the carbon capsules can impede the growth and aggregation of the core Co_9_S_8_ NPs while buffering the volume change upon the electrochemical reaction, which ensures a stable SEI film and alleviates the capacity fading against extended cycling.

### Sodium-Ion Storage Performance in Half Cells

**Fig. 3:**
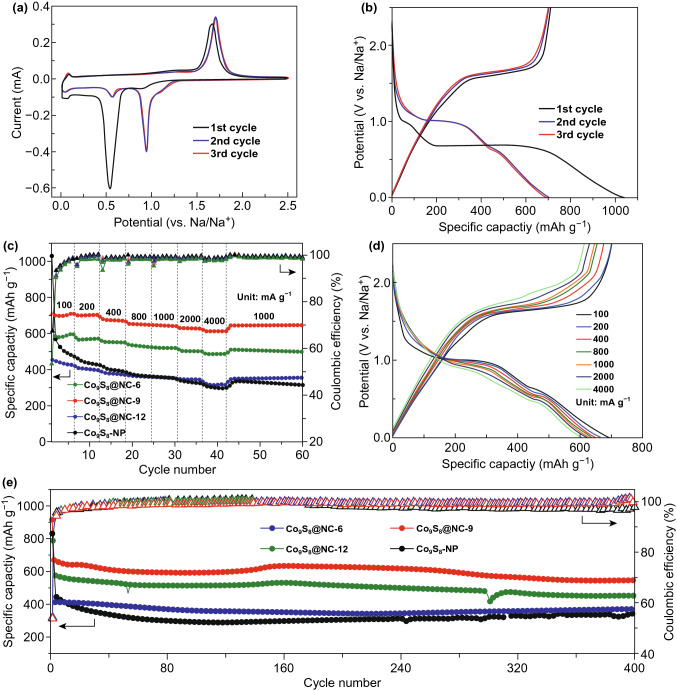
Electrochemical performance of the Co_9_S_8_@NC electrodes. **a** CV profiles of Co_9_S_8_@NC-9 electrode for the initial three cycles. **b** Galvanostatic discharge–charge profiles of Co_9_S_8_@NC-9 at 100 mA g^−1^. **c** Rate performance and corresponding **d** discharge–charge profiles at various current densities from 100 to 4000 mA g^−1^. **e** Long-term cycling stability at a high current density of 1000 mA g^−1^

To evaluate the Co_9_S_8_@NC as anode for NIBs, the electrochemical performance was tested in coin-type half cells. Figures 3a and S6a, b show the CV curves of Co_9_S_8_@NC electrodes for the initial three cycles. In the CV profile of Co_9_S_8_@NC-9, the initial reduction process shows peaks between 0.15 and 1.0 V, which corresponds to the sodiation of both carbon and Co_9_S_8_, along with the decomposition of electrolyte and the formation of the SEI layer. In particular, the peak located at 0.9 V corresponds to the interaction of Co_9_S_8_ with sodium ions to form a Na_2−*x*_Co_9_S_8_ phase, while the peak at 0.55 V is related to Na_2-*x*_Co_9_S_8_ transforming to Co and Na_2_S via conversion reaction [4], which will be illustrated later by TEM and in situ XRD results. In the subsequent cycles, the new cathodic peak at 0.95 evolved instead, which are related to the reaction between Na_2−*x*_Co_9_S_8_ and sodium ions. During the anodic scan, the oxidation peak located at 1.67 V is due to the desodiation process. Moreover, there are a pair of reversible peaks located at ~ 0.1 V for both cathodic and anodic scans, which are due to the intercalation/deintercalation of solvated sodium ions into/from nano-voids formed by the disordered carbon nanosheets [39].

Accordingly, the galvanostatic discharge–charge profiles of Co_9_S_8_@NC electrodes are shown in Figs. 3b and S6c, d. The long discharge plateau in the first cycle is related to the sodiation process and SEI formation, accordant with the CV profiles. The initial discharge/charge capacities of Co_9_S_8_@NC-6, Co_9_S_8_@NC-9, and Co_9_S_8_@NC-12 electrodes were 568/430, 1034/709, and 807/601 mAh g^−1^ at 100 mA g^−1^, respectively. The first irreversible capacity is attributed to the partial reductive decomposition of the electrolyte and the SEI formation. The overlapping of the discharge–charge profiles as well as the CV curves after the first cycle of the Co_9_S_8_@NC-9 is an indication of the highly reversible reaction of the electrode materials with sodium ions. In addition, it should be noticed that excessive carbon content in the Co_9_S_8_@NC-6 sample led to inferior performance, which might stem from the low capacity of carbon.

Impressively, the Co_9_S_8_@NC-9 electrode shows an extraordinary rate performance by achieving capacities of 705, 701, 675, 652, 645, 629, and 613 mAh g^−1^ at current densities of 100, 200, 400, 800, 1000, 2000, and 4000 mA g^−1^, respectively, as shown in Fig. 3c and d, which are better than Co_9_S_8_@NC-6, Co_9_S_8_@NC-12, and Co_9_S_8_-NP electrodes (Figs. 3c and S6e, f). Importantly, after successive 43 cycles at different current densities, the capacity of Co_9_S_8_@NC-9 was retained to 645 mAh g^−1^ when the current density was changed to 1000 mA g^−1^. The excellent rate performance of Co_9_S_8_@NC-9 was attributed to the fast charge transport kinetics throughout the electrode, likely due to both material integrity against the volumetric expansion as well as the high electron conductivity from the rational-designed structure with multi-functional electrochemical active components. Moreover, the cycling performance of the Co_9_S_8_@NC-6, Co_9_S_8_@NC-9, Co_9_S_8_@NC-12, and Co_9_S_8_-NP electrodes were investigated, as shown in Fig. 3e. The Co_9_S_8_@NC-9 electrode shows no virtually capacity fading at a high rate of 1000 mA g^−1^ over 400 cycles with a high Coulombic efficiency of ~ 99.8%, indicating a high reversible reaction with sodium ions and the structural stability of the electrode material. The retained capacity of Co_9_S_8_@NC-6, Co_9_S_8_@NC-9, Co_9_S_8_@NC-12, and Co_9_S_8_-NP electrodes after 500 cycles were 556, 458, 374, and 331 mAh g^−1^, corresponding to the capacity retention of 82, 78, 81, and 69%, respectively. It is noted that the Co_9_S_8_@NC electrodes exhibit better cycling performance and rate capability than that of the Co_9_S_8_-NP electrode, which could be attributed to the introduction of carbon encapsulation as well as the nitrogen doping. It can also be concluded that the Co_9_S_8_@NC-9 electrode with an intact core–shell structure, hierarchical pores on the carbon layers and a relatively large surface area is the optimised choice in this work by providing high capacity, excellent rate capability and good stability against long-term cycling.

### Sodium-Driven Structural and Compositional Changes

**Fig. 4:**
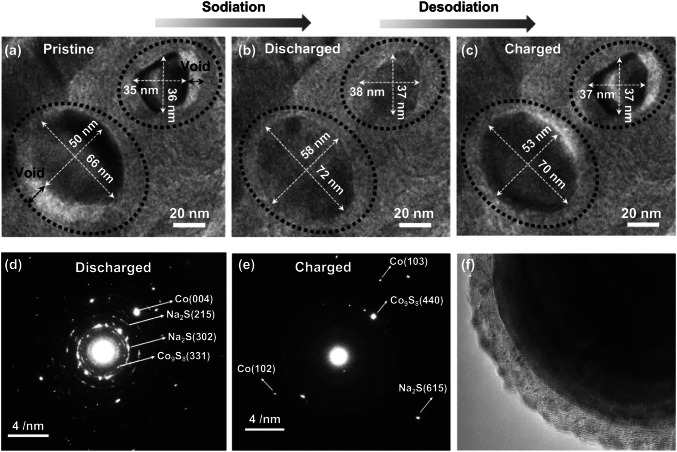
a-c *In situ* TEM investigation of structural changes of the Co_9_S_8_@NC electrode during varied sodiation depths. SAED patterns the Co_9_S_8_@NC electrode at **d** sodiated to 0.01 V and **e** desodiated to 2.5 V illustrating the compositional changes. **f** HRTEM images of Co_9_S_8_@NC electrode after long-term cycling after 400 cycles

To understand the structural merits of the Co_9_S_8_@NC-9 associated with this fast and highly stable reaction, in situ and ex situ TEM techniques were performed to optically probe the composition and structure of the Co_9_S_8_@NC-9 electrode during electrochemical testing. The volume changes of the Co_9_S_8_@NC-9 due to sodiation/desodiation were captured of the initial, sodiated and desodiated stages, shown in Fig. 4a–c. In pristine electrode, a core–shell structure with a hollow interior between the Co_9_S_8_ core and the carbon shell (in white circles, Fig. 4a) can be observed. During the consecutive sodiation, the interior voids were gradually filled by the expanded Co_9_S_8_ core, clearly illustrating that the Co_9_S_8_ electrode material expanded upon sodiation and ultimately confined by the carbon shells. To measure the volume expansion, two core Co_9_S_8_ NPs with diameters of ≈ 50 nm in width and ≈ 66 nm in length, and ≈ 35 nm in width and ≈ 36 nm in length, respectively, were selected as indicated by the prominent markers and arrows. After sodiation, the two positioning arrows placed at the markers expanded to ≈ 58 and 72 nm for the large NP and to ≈ 38 and 37 nm for the small NP, respectively, giving a volume expansion of ≈ 46 and 21%, respectively. These values are much smaller than the observed volume expansion in a previous study, i.e., 120.8% expansion of Co_9_S_8_ upon sodiation [7], due to the confinement effect from the graphitic carbon layers. Moreover, the sodiated Co_9_S_8_@NC-9 presented neither fracture of carbon layers nor degradation of Co_9_S_8_ NPs, indicating the successful confinement of Co_9_S_8_ NPs by the mechanically robust carbon layers. Figure S7 shows ex situ HRTEM image of the sodiated electrode, displaying plenty of nanograins of about 5–10 nm in size. The SAED pattern of the sodiated region (Fig. 4d) confirms the formation of Na_2_S and Co as conversion products. After desodiation, the volume of Co_9_S_8_ NPs returned to a slight expansion of 19 and 15%, respectively. The SAED pattern demonstrates that the conversion products of Co and Na_2_S returned to Co_9_S_8_ with tiny residues, as shown in Fig. 4e. Moreover, Figs. 4f and S8 show HRTEM images of the long-cycled Co_9_S_8_@NC-9 electrode, verifying that Co_9_S_8_ NPs were still restricted by carbon shells and carbon layers were well preserved after long-term cycling. The low volume variation, as well as the excellent structural stability of the Co_9_S_8_@NC-9 electrode, provides solid evidence for its excellent cycling performance in real batteries.

**Fig. 5:**
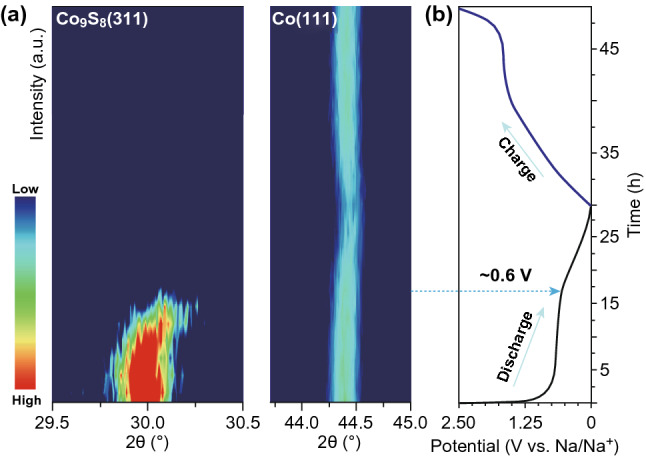
*Operando* XRD patterns collected at various states of the initial discharge/charge process of a Co_9_S_8_@NC-9/sodium electrochemical cell. Contour plots of the diffraction peak evolution of Co_9_S_8_ (311) and Co (111) planes. The corresponding galvanostatic discharge–charge profiles at a current density of 20 mA g^−1^ from the open circuit potential to 0.01 V

The sodium storage mechanism of the Co_9_S_8_@NC-9 electrode was further confirmed by means of *operando* XRD at various sodiated and desodiated stages of the first cycle (Fig. 5a). The corresponding galvanostatic discharge–charge profile at a current density of 20 mA g^−1^ is shown in Fig. 5b. During the first discharging, the (311) reflection of Co_9_S_8_ shifted gradually towards a higher 2*θ* degree (smaller *d*-spacing), which is likely due to the reinforced electrostatic attraction between the inserted sodium ions and Co_9_S_8_ lattice [40]. Subsequently, the (311) peak weakened and eventually vanished after 0.6 V, indicating the ongoing structural transformation from Na_*x*_Co_9_S_8_ to Co and Na_2_S. Due to the small nanocrystal size (~ 2–5 nm shown in TEM images) of the conversion products, the reflections of the newly formed Na_2_S and Co could be broad and become part of the background of the XRD patterns. Meanwhile, a gradual shift of the Co (111) reflection towards a higher angle is observed, indicating a slight lattice contraction during the sodiation process. After sodium ions retrieved from the electrode, the Co (111) peak is fully recovered to its pristine state. The reversible interaction of Co_9_S_8_ with sodium ions is benefiting from the well-retained structure that restrains the loss of active components during conversion reaction.

### Electrochemical Performance in a Sodium-Ion Capacitor

**Fig. 6:**
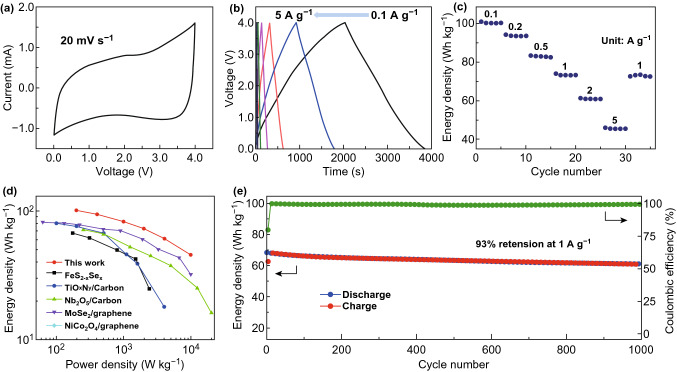
Electrochemical performance of the Co_9_S_8_@NC-9//CG NIC cells. **a** The CV curves of the Co_9_S_8_@NC-9//CG NIC full cell. **b** Galvanostatic charge–discharge profiles and **c** rate performance at various current densities from 0.1 to 5 A g^−1^. **d** Ragone plots of the Co_9_S_8_@NC-9//CG NIC cell and the comparison with other reported NIC systems. **e** Cycling performance of a Co_9_S_8_@NC-9//CG NIC at a rate of 1 A g^−1^

To demonstrate the feasibility of using Co_9_S_8_@NC-9 for high-power energy storage applications, we fabricated a Swagelok-type full cell sodium-ion capacitor (NIC) using the pre-sodiated Co_9_S_8_@NC-9 as the negative electrode, paired with the cellulose-derived porous carbon/graphene oxide composites (CG) as the ion adsorption/desorption positive electrode. The structure characterisation of the CG positive electrode is shown in Fig. S9. The CV test of Co_9_S_8_@NC-9 and CG electrodes at various scan rates was performed in sodium half cells, as shown in Fig. S10. When assembled for a NIC full cell, the CV curve of the Co_9_S_8_@NC-9//CG NIC exhibits a typical capacitive charge storage behaviour, as shown in Fig. 6a, indicating that Co_9_S_8_@NC-9 electrode could be a suitable anode for NICs. Figure 6b shows the galvanostatic charge–discharge profiles of the Co_9_S_8_@NC//CG NIC at various current densities from 0.1 to 5 A g^−1^. The nearly linear profiles at various current rates are indicative of the fast charge storage kinetics. As shown in the Ragone plots of NIC full cells in Fig. 6d, the as-assembled NIC delivered a superior energy density of 101.4 Wh kg^−1^ at a power density of ~ 200 W kg^−1^ and maintained an energy density of 45.8 Wh kg^−1^ at a high power density of ~ 10,000 W kg^−1^, demonstrating an extraordinary high rate characteristic. When compared with various recently reported high-performance NICs [41–44], the Co_9_S_8_@NC-9//CG NIC device shows superior high energy and high power performance. Moreover, the NIC shows a high capacity retention of 93% and high Coulombic efficiency of about 99.9% over 1000 cycles at 1 A g^−1^ (Fig. 6e), manifesting the advantages of hybrid devices with both highly reversible sodium-ion storage capability and good cycling stability. The above results indicate that the as-designed Co_9_S_8_@NC electrode with a unique structure would open the avenue to advanced high-performance NICs.

## Conclusions

Herein, we report a high electrochemical performance composite anode with cobalt sulphide nanoparticles encapsulated by a spherical carbon shell with nitrogen doping for sodium-ion storage. The high conductivity of carbon and the core–shell-like structure of the electrode material contributed to the enhancement in the electrochemical properties of the composite electrode in a synergistic manner. In-depth investigation using in situ TEM confirmed the effect of carbon buffering the volume change. Further, the carbon shell was well preserved after repeated cycling. High capacity and shelf-life along with scalability make the present material attractive as next-generation sodium-ion battery anode material. This work could be translated to other transition metal chalcogenides using such material design to realise inexpensive and durable electrode materials for practical application in batteries.

## Electronic supplementary material

Below is the link to the electronic supplementary material.

Supplementary material 1 (PDF 1136 kb)
