# Supplementary material for: Stabilising Cobalt Sulphide Nanocapsules with Nitrogen-Doped Carbon for High-Performance Sodium-Ion Storage
Source: Nanomicro Lett. 2020 Feb 12;12:48. doi: 10.1007/s40820-020-0391-9 (PMC7770835; doi:10.1007/s40820-020-0391-9)
Supplement: Supplementary file 1 — Supplementary material 1 (PDF 1136 kb) [file 40820_2020_391_MOESM1_ESM.pdf]

Supporting Information for

Stabilising Cobalt Sulphide Nanocapsules with Nitrogen-Doped Carbon for High-Performance Sodium-Ion Storage

Yilan Wu[1, #], Rohit R. Gaddam[1, #], Chao Zhang[2], Hao Lu[1], Chao Wang[3], Dmitri Golberg[2], Xiu Song Zhao[1, 3, *]

[1]School of Chemical Engineering, The University of Queensland, St Lucia, Brisbane, QLD 4072, Australia

[2]School of Chemistry, Physics and Mechanical Engineering, Science and Engineering Faculty, Queensland University of Technology, Brisbane, QLD, 4001, Australia

[3]Institute of Materials for Energy and Environment, College of Materials Science and Engineering, Qingdao University, 308 Ningxia Road, Qingdao 266071, People's Republic of China

[#]Yilan Wu and Rohit R. Gaddam contributed equally to this work

*Corresponding author. E-mail: george.zhao@uq.edu.au

S1 Characterization

X-ray diffraction (XRD) patterns were collected on a Bruker D8 Advance X-ray diffractometer (Cu Kα radiation, λ = 1.54056 Å). *Operando* XRD patterns were acquired on a Rigaku SmartLab X-ray diffractometer with Cu Kα radiation (λ = 1.5406 Å). The electrode was loaded in the XRD cell with a Beryllium window in an argon-filled glovebox. The XRD cell unit was connected to a SP150 (Biologic, France) single-channel potentiostat for charging/discharging during XRD measurements. The electrochemical measurements were carried out at a current density of 20 mA g^{-1} and at a XRD scanning rate of 0.5° (2θ) min^{-1}. The morphology of samples was examined on a field-emission scanning electron microscope (FESEM, JEOL 7001) at 10 kV. Transmission electron microscopy (TEM) and high-resolution TEM (HRTEM) measurements were conducted on a JEOL-JEM-2100F microscope equipped with an energy dispersive X-ray (EDX). For in-situ TEM observation, a tungsten tip covered with Co_9S_8@NC-9 sample was loaded on the TEM-STM holder as an electrode. Sodium metal with a grown Na_2O layer was mounted on a piezo-driven biasing probe to serve as a Na source, and a thin Na_2O layer served as the solid electrolyte, as shown in Fig. S1. The sample was brought into contact with the Na_2O/Na particles, and a high voltage bias of -5V was applied by means of potentiostate to drive the sodiation reaction. The potential was larger than that used in the tests of sodium ion half-cells due to the necessity to drive the sodium ions through the solid electrolyte and carbon layer. X-ray photoelectron spectroscopy (XPS) spectra were acquired on a Kratos Axis photoelectron spectrometer equipped with an Al (Kα 1486.6 eV) radiation. Nitrogen adsorption/desorption isotherms were measured on a Tristar II 3020 instrument at 77 K. Samples were degassed at 150 °C overnight before measurements of nitrogen adsorption.

S2 Electrochemical Measurements

Electrodes were prepared by mixing an active material, carbon black and polyvinyldine fluoride (PVDF) at a mass ratio of 7:2:1 in *N*-methyl pyrrolidine (NMP) solvent under magnetic stirring to form a slurry, which was subsequently casted onto a current collector with a copper foil for the Co_9S_8@NC electrodes and an aluminium foil for the CG electrode using a doctor blade. The electrode was dried at 80 °C overnight in a vacuum oven.

Sodium-ion batteries were assembled using the working electrode, glass fibre separator (GF/D, Whatman) and sodium metal counter electrode in 2032-type coin cells. Sodium-ion capacitors (NICs) were assembled using Co_9S_8@NC-9 as negative electrode and CG as positive electrode. The mass loadings of the active materials on the electrodes were about 0.8~1.0 mg cm^{-2} for Co_9S_8@NC electrodes, and about 3.0~5.0 mg cm^{-2} for CG electrodes, respectively. To satisfy the charge balance of the two electrodes ($Q_+ = Q_-$, namely, $m_+q_+ = m_-q_-$, where Q is the capacity, q is the specific capacity, and m is the mass of the active materials)[1], the mass ratio of the active materials of CG:Co_9S_8@NC-9 was carefully adjusted from 1:3 to 1:6 due to the specific capacity difference of the positive and negative materials. When the mass ratio of the active materials of CG: Co_9S_8@NC-9 was about 1:5, the as-fabricated NIC full cell exhibited optimum energy and power performance. Before assembling NICs, the Co_9S_8@NC-9 electrode was pre-cycled at 0.1 A g^{-1} for 3 cycles in a sodium half-cell. The electrolyte was comprised of 1.0 mol L^{-1} sodium hexafluorophosphate ($NaPF_6$) in diglyme solvent. The electrochemical properties of the electrodes were determined by using cyclic voltammetry (CV) on a VPM3 (Biologic, France) and galvanostatic charge-discharge measurements on a Neware battery measurement system (CT3008).

The energy density and power density of the NIC full cells were calculated by using Eqs. S1 and S2:

$$E = I * \int V dt \qquad \text{(S1)}$$

$$P = E/t \qquad \text{(S2)}$$

Where E (Wh kg^{-1}) is the energy density, P (W kg^{-1}) is the power density, I (A g^{-1}) is the constant current density, V (V) is the working voltage, and t (s) is the discharge time. Note that the current density was obtained based on the total mass of both the positive and negative active materials.

S3 Supplementary Figures and Table

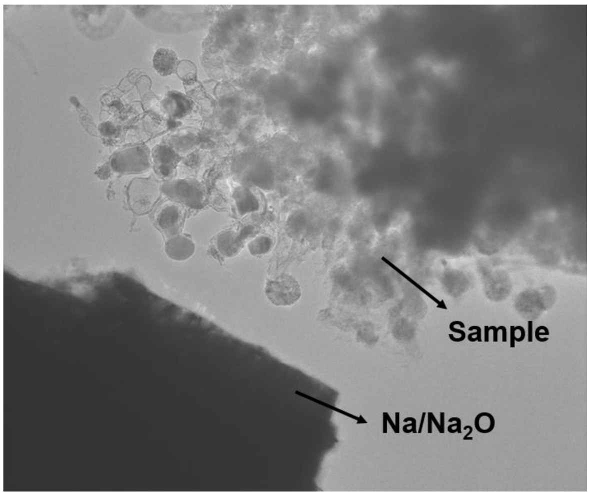

Fig. S1 In-situ TEM image of the configuration

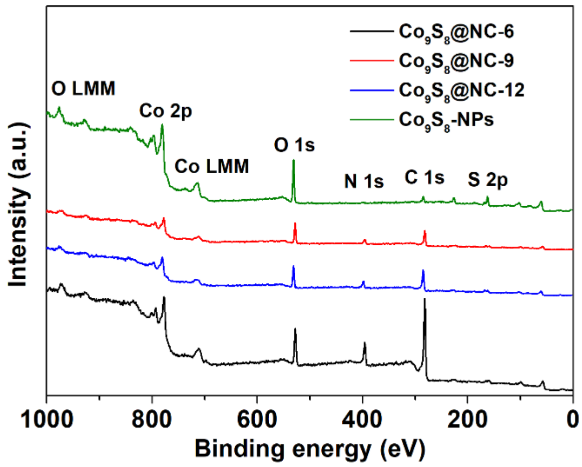

Fig. S2 XPS survey spectrum of the as-prepared Co_9S_8@NC composites and Co_9S_8-NPs

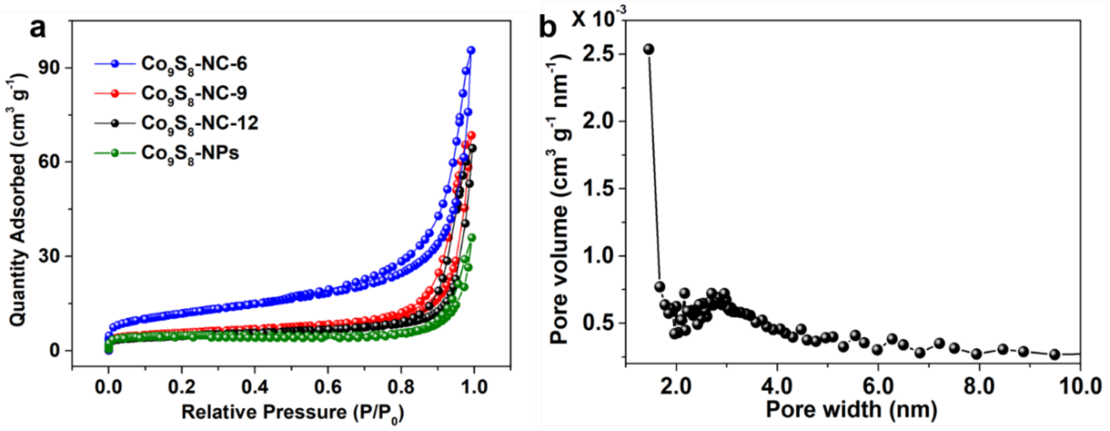

Fig. S3 (a) Nitrogen adsorption-desorption isotherms of the Co_9S_8@NC samples. **(b)** The pore size distribution of Co_9S_8@NC-9 calculated using the BJH method

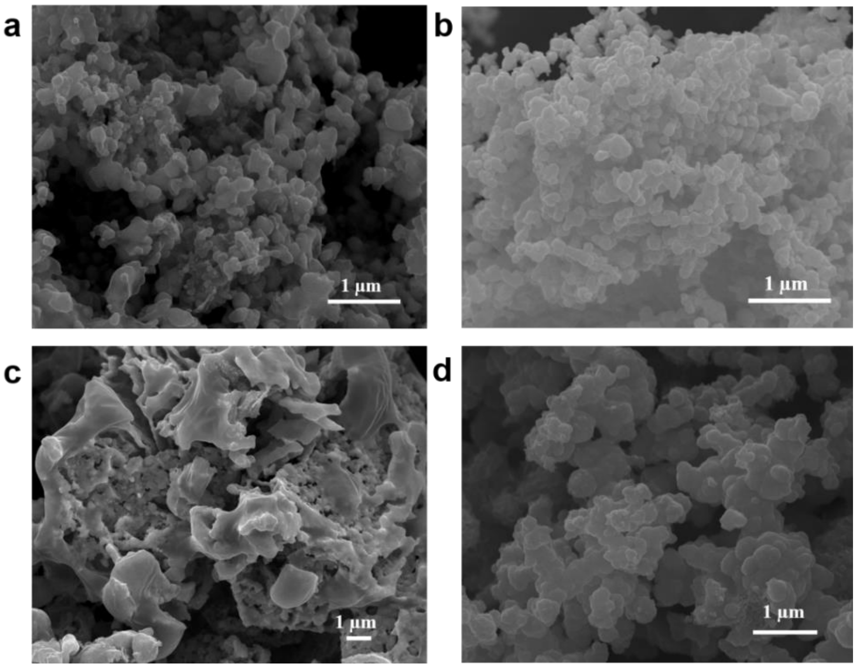

Fig. S4 FESEM images of **(a)** Co_9S_8@NC-6, **(b)** Co_9S_8@NC-9, **(c)** Co_9S_8@NC-12, and **(d)** Co_9S_8-NP

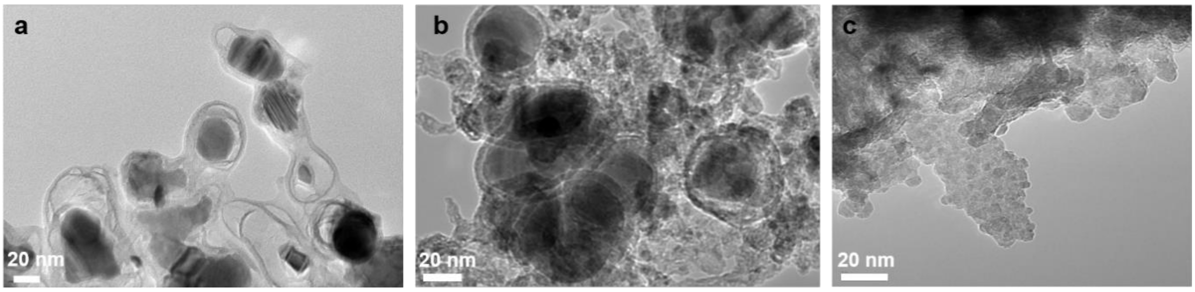

Fig. S5 TEM images of **(a)** Co_9S_8@NC-6, **(b)** Co_9S_8@NC-12, and **(c)** Co_9S_8-NP

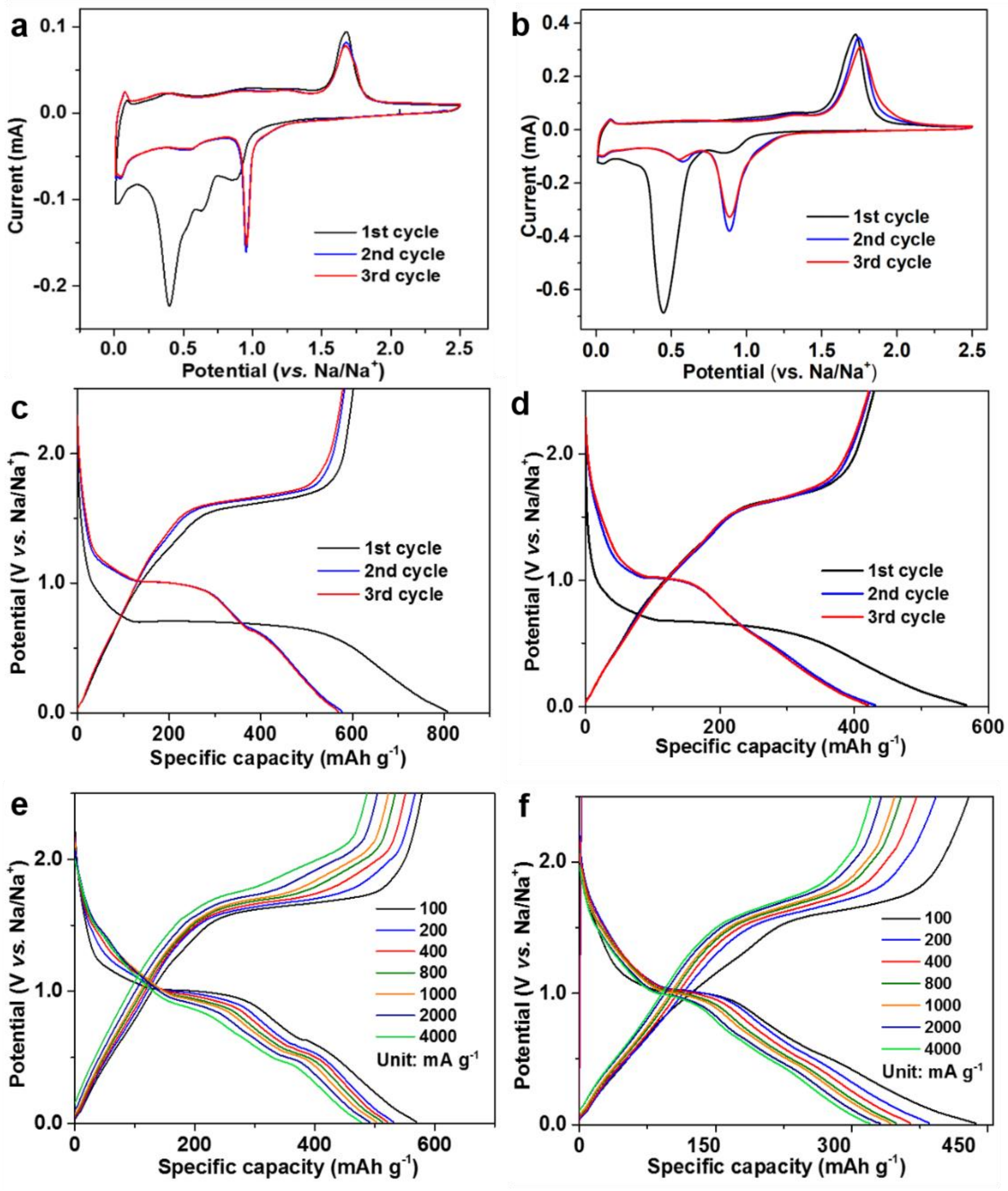

Fig. S6 CV profiles of **(a)** Co_9S_8@NC-6 and **(b)** Co_9S_8@NC-12 electrode for the initial three cycles. Galvanostatic discharge-charge profiles of **(c)** Co_9S_8@NC-6 and **(d)** Co_9S_8@NC-12 at 100 mA g^{-1}. Discharge-charge profiles at various current densities from 100 to 4000 mA $g^{-1}$of **(e)** Co_9S_8@NC-6 and **(f)** Co_9S_8@NC-12

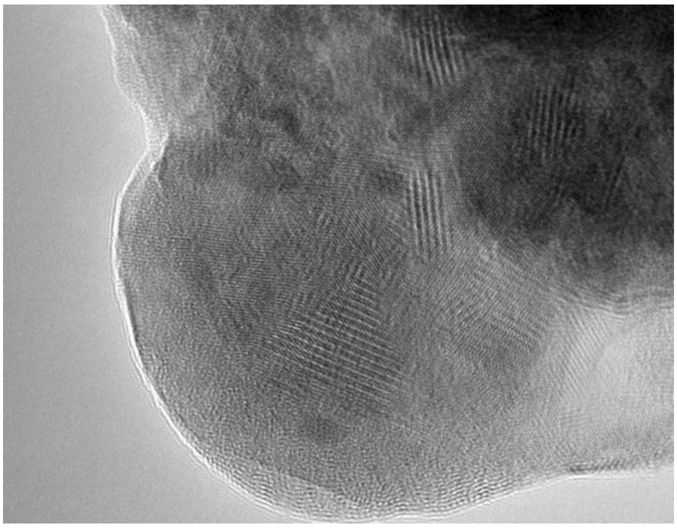

Fig. S7 HRTEM image of Co_9S_8@NC-9 electrode after sodiation

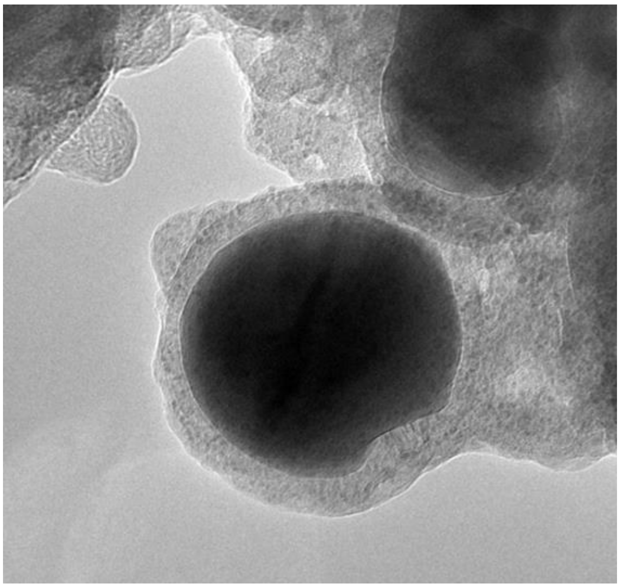

Fig. S8 TEM image of Co_9S_8@NC-9 electrode after long-term cycling

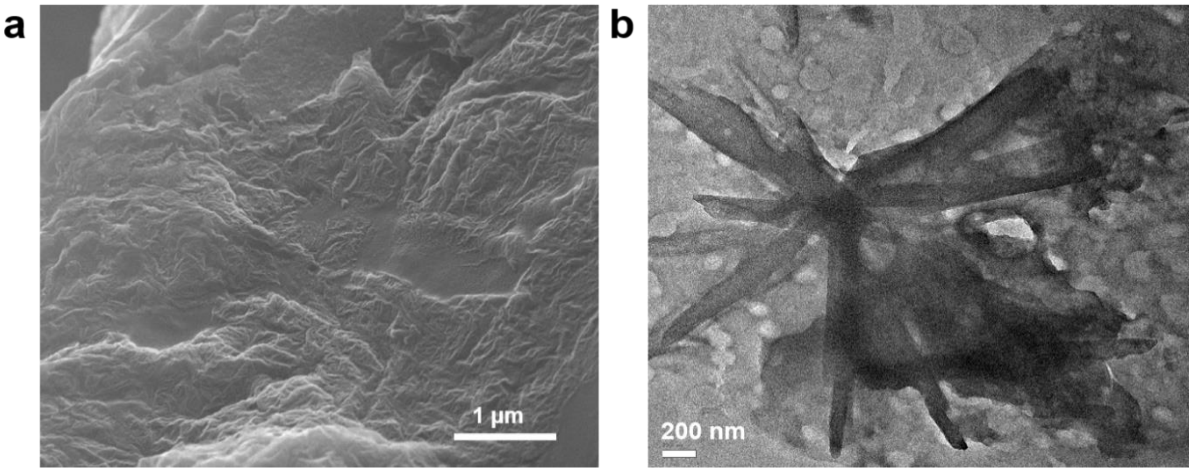

Fig. S9 (a) FE-SEM and **(b)** TEM images of the cellulose-derived porous carbon/graphene oxide composites (CG)

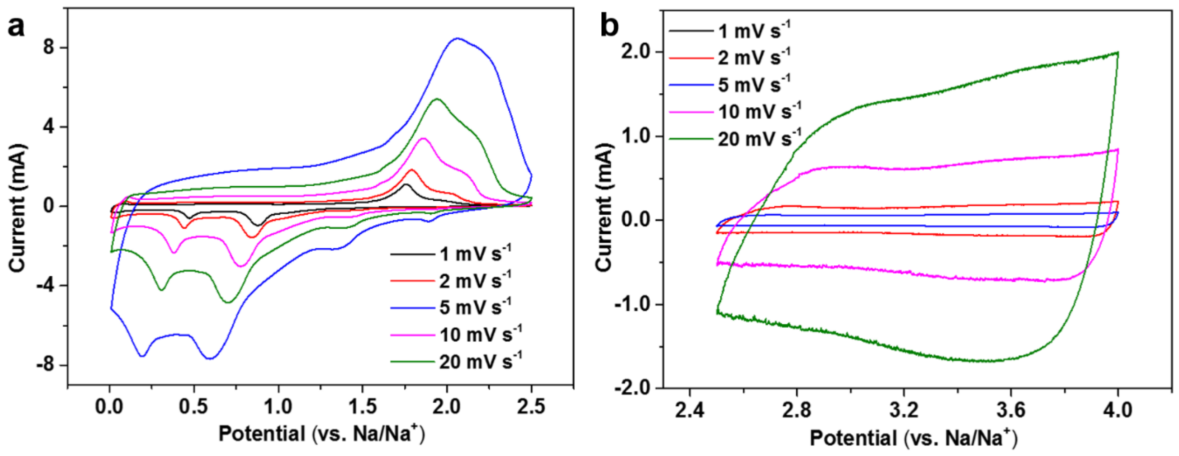

Fig. S10 CV profiles of **(a)** Co_9S_8@NC-9 and **(b)** CG at various scan rate in sodium half-cells

Table S1 Surface elemental composition of the as-prepared Co_9S_8@NC composites and Co_9S_8-NPs according to the XPS survey measurements

**Materials**	**Co (at%)**	**S (at%)**	**C (at%)**	**N (at%)**	**O (at%)**
Co_9S_8@NC-6	5.92	1.57	69.68	11.88	10.95
Co_9S_8@NC-9	7.18	5.01	55.99	13.27	18.55
Co_9S_8@NC-12	8.33	5.11	52.47	11.92	22.17
Co_9S_8-NPs	28.19	12.86	17.38	-	28.19

Supplementary References

[S1]C. Zhan, W. Liu, M. Hu, Q. Liang, X. Yu, Y. Shen, R. Lv, F. Kang, Z.-H. Huang, High-performance sodium-ion hybrid capacitors based on an interlayer-expanded MoS_2/rGO composite: Surpassing the performance of lithium-ion capacitors in a uniform system. NPG Asia Mater. **10**(8), 775-787 (2018). https://doi.org/10.1038/s41427-018-0073-y
